# Moving upstream: healthcare partnerships addressing social determinants of health through community wealth building

**DOI:** 10.1186/s12889-023-16761-x

**Published:** 2023-09-19

**Authors:** Geoffrey M. Gusoff, David Zuckerman, Bich Ha Pham, Gery W. Ryan

**Affiliations:** 1 National Clinician Scholars Program & Department of Family Medicine, University of California, Los Angeles, 1100 Glendon Ave, Suite 900, Los Angeles, CA 90024 USA; 2 Healthcare Anchor Network, 2202 18th St. NW, Suite 317, Washington, DC 20009 USA; 3 Department of Health Systems Science, Kaiser Permanente Bernard J. Tyson School of Medicine, 100 South Los Robles Avenue, Pasadena, CA 91101 USA

**Keywords:** Social determinants of health, Housing and health, Community benefits, Health and wealth, Community economic development

## Abstract

**Background:**

Healthcare-based interventions addressing social needs such as food and housing generally fail to impact the upstream wealth and power inequities underlying those needs. However, a small number of US healthcare organizations have begun addressing these upstream inequities by partnering with community wealth building initiatives. These initiatives include community land trusts, resident-owned communities, and worker cooperatives, which provide local residents ownership and control over their housing and workplaces. While these partnerships represent a novel, upstream approach to the social determinants of health, no research has yet evaluated them.

**Methods:**

To assess the current state and key aspects of healthcare-community wealth building partnerships, we conducted a multiple case study analysis using semi-structured interviews with thirty-eight key informants across ten partnerships identified through the Healthcare Anchor Network. To analyze the interviews, we used a two-stage coding process. First, we coded responses based on the phase of the intervention to which they corresponded: motivation, initiation, implementation, or evaluation. Then we assessed responses within each aspect for common themes and variation on salient topics.

**Results:**

Partnerships were generally motivated by a combination of community needs, such as affordable housing and living wage jobs, and health system interests, such as workforce housing and supply chain resilience. Initiating projects required identifying external partners, educating leadership, and utilizing risk mitigation strategies to obtain health system buy-in. Implementation took various forms, with healthcare organizations providing financial capital in the form of grants and loans, social capital in the form of convening funders and other stakeholders, and/or capacity building support in the form of strategic planning or technical assistance resources. To evaluate projects, healthcare organizations used more process and community-level metrics rather than metrics based on individual health outcomes or returns on investment. Based on best practices from each partnership phase, we provide a roadmap for healthcare organizations to develop effective community wealth building partnerships.

**Conclusions:**

Assessing healthcare partnerships with community wealth building organizations yields key strategies healthcare organizations can use to develop more effective partnerships to address the upstream causes of poor health.

## Background

A potential paradigm shift is quietly emerging in the way US healthcare organizations address the social determinants of health. In the wake of the Affordable Care Act’s requirement of Community Health Needs Assessments for non-profit hospitals, [1] the growing role of value-based payments, [2] and a proliferation of evidence linking social needs to health, it is increasingly common in the US for healthcare organizations to assess for social needs, offer referrals, and even provide patients direct food, housing, or job-training support [3]. While these interventions can provide crucial assistance for patients facing toxic exposures like housing or job insecurity, they do little to address the upstream wealth and power inequities that underlie those insecurities and are themselves major drivers of poor health [4–9]. That is beginning to change.

A small number of US healthcare organizations have begun incorporating community wealth building (CWB) strategies into their approaches to addressing the social determinants of health. Community wealth building is defined by The Democracy Collaborative – the organization that coined term in 2005 – as “an economic development model that transforms local economies based on communities having direct ownership and control of their assets” [10]. The CWB approach addresses housing and job insecurity in an upstream way by changing the ownership and control of land and businesses at the local level within particular neighborhoods, towns, or municipalities. Three increasingly popular models that embody this approach are community land trusts, resident-owned communities, and worker cooperatives.

Community land trusts (CLTs) are non-profit organizations that acquire housing and then sell it to low- and moderate-income households at an affordable price in exchange for a guarantee that the price will remain affordable upon resale [11]. The CLT board is democratically governed by residents of the CLT and other local stakeholders. The CLT model has been shown to provide permanent affordability and wealth-building for residents while preventing displacement [12, 13]. There are currently 225 land trusts in the US with approximately 12,000 units of housing [14].

In a similar model, resident-owned communities (ROCs) are groups of mobile home owners who cooperatively purchase the land under their homes instead of paying rent to an outside owner [15]. Like CLTs, the ROC model promotes long-term affordability and stability for low- and moderate-income households [16]. There are currently 1,065 resident-owned communities in the US, representing 2.3% of all manufactured home communities [16]. CLTs and shared equity housing models similar to ROCs have also grown significantly internationally in both the Global North and Global South [17–19].

Worker cooperatives are businesses that are owned and democratically governed by their workers. Compared to traditional businesses in the same industries, employment at a worker-owned cooperative has been associated with improvements in wages, benefits, work conditions, job stability, and wealth-building opportunities [20–22]. There are approximately 900 worker cooperatives in the US employing about 10,000 workers [23]. While this is a very small portion of the US labor force, in several international contexts worker cooperatives have demonstrated a capacity to scale up and play a major role in regional economies, particularly in the Basque region of Spain and the Emilia Romagna region of Italy [24, 25].

U.S. healthcare organization partnerships with CWB organizations like CLTs, ROCs, and worker cooperatives have been reported in the gray literature but have not been systematically studied. Several of these partnerships involve healthcare organizations that are members of the Healthcare Anchor Network (HAN), a nonprofit organization that facilitates a “national collaboration of 70 + leading healthcare systems building more inclusive and sustainable local economies” [26]. HAN helps facilitate community partnerships for healthcare organizations in several ways, including identifying best practices and providing technical assistance and strategic planning resources. This study, a collaboration between two academic researchers (GG and GR) and two members of HAN’s leadership (DZ and BHP), aims to identify and assess existing healthcare organization partnerships with CWB initiatives and to provide a roadmap for healthcare organizations interested in pursuing similar partnerships.

## Methods

To fulfill these aims, we employed a multiple case study approach to more deeply understand each partnership, while also identifying common themes and variation among partnerships [27]. To collect data about each partnership, we conducted semi-structured interviews with key informants [28].

We employed a two-level strategy to select partnerships and participants within each partnership. First, we identified all relevant partnerships through HAN’s membership network and reviews of academic and gray literature. We included in the multiple case study all identified partnerships in which a healthcare organization funded, invested in, or purchased from a CLT, ROC, or worker cooperative. Second, within each partnership we identified key informants from both the healthcare organization and CWB organization involved in the partnership. We identified healthcare organization key informants through HAN’s network and community organization key informants either through snowball sampling from the healthcare organization informants or through the community organizations’ websites. In identifying key informants, we sought to speak with individuals most familiar with the partnership from its inception to the present and those who could offer a variety of perspectives to provide both a depth and breadth of information.

We conducted semi-structured interviews with the key informants that explored four domains of each partnership: motivation, initiation, implementation, and evaluation. The list of domains was adapted from Haldane et al.’s framework for healthcare-community collaborations based on input from HAN practitioners [29]. The motivation domain assessed healthcare organizations’ rationales for engaging in the partnership, the *why* of the partnership. The initiation domain assessed the ways in which healthcare organizations started the partnership, the *how* of the partnership. The implementation domain assessed the healthcare organization’s roles and resources involved in the partnership, the *what* of the partnership. Finally, the evaluation domain assessed the outcomes of the partnership, answering the *what impact* question regarding the partnership. We assessed each domain through a series of open-ended questions. For example, we assessed initiation by asking “walk me through the steps taken to start this project from the earliest stage.” Interviews were conducted in English over Zoom between October 2021 and April 2022.

Interview analysis consisted of a two-stage coding approach. First, we coded components of the transcripts according to the four domains of the partnership. Then, within each domain, we assessed the range of responses and common themes among responses, focusing on elements that would be most relevant to healthcare organizations seeking to build similar partnerships, in accordance with our study aims [30]. The academic researchers on the study team conducted the initial interview coding (GG) and review of the coding (GR), and the HAN practitioner members of the team (DZ and BHP) provided input on the relevance of responses and themes to healthcare organizations. Based on the final coding, the academic researchers drafted a practitioner “roadmap” comprised of relevant themes from the cases and their main implications for future practice. The roadmap was then modified based on additional input from the HAN practitioner team members.

## Results

We identified a total of 10 relevant US partnerships – 6 with CLTs, 1 with a ROC, and 3 with worker cooperatives, all of which agreed to participate in the study. For eight of the partnerships identified, the healthcare organization partner was a HAN member. Of the remaining partnerships with non-HAN members, one was identified by HAN and the other was identified in the gray literature. We conducted 29 interviews with a total of 38 key informants from a variety of roles and institutions. For every partnership, we interviewed at least one key informant from the healthcare organization and at least one key informant from the CWB organization partner, except for one CLT partnership for which we were unable to reach an appropriate representative from the healthcare organization. Table 1 summarizes the characteristics of the sample of partnerships and key informants.

**Table 1:** List of partnership cases and key informant affiliations

Partnership Members	Location	CWB Intervention	Healthcare Organization Key Informants	CWB Organization Key Informants
Bon Secours Richmond Health System – Maggie Walker Community Land Trust	Richmond, Virginia	Community Land Trust	4	1
Bon Secours St. Francis Health System – Sterling Land Trust	Greenville, South Carolina	Community Land Trust	2	2
Mayo Clinic – First Homes Community Land Trust	Greater Rochester Area, Minnesota	Community Land Trust	0	2
Nationwide Children’s Hospital – Central Ohio Community Land Trust	Columbus, Ohio	Community Land Trust	2	2
Saint Alphonsus Health System – LEAP Housing	Boise, Idaho	Community Land Trust	1	1
UMass Memorial Medical Center – Worcester Common Ground	Worcester, Massachusetts	Community Land Trust	6	1
Dartmouth Health – ROC-NH	New Hampshire (statewide)	Resident-Owned Community	1	2
Baystate Health – Wellspring Cooperative	Springfield, Massachusetts	Worker Cooperative	1	1
Cleveland Clinic & University Hospitals – Evergreen Cooperatives	Cleveland, Ohio	Worker Cooperative	3	4
Kaiser Permanente – Obran & Project Equity	Nationwide	Worker Cooperative	1	1
**Total**			**21**	**17**

*CWB *Community wealth building

The healthcare organization partners were all non-profit entities that were themselves, or were affiliated with, larger “health systems” – regional or national networks of hospitals, clinics, and other care sites. The CWB organizations ranged from recently formed organizations exclusively focused on CLTs, ROCs, or worker cooperatives to more established, multi-faceted entities in which their CWB interventions were part of broader work in housing, community financing, or other areas. The CLT partners were themselves administrators of the CLTs while the ROC and worker cooperative partners were organizations that develop ROCs and worker cooperatives, which generally become separate entities. Nearly all of the CWB organizations were non-profits with the exception of Obran, which was organized as a private conglomerate of its worker cooperative members.

We found that CLT and ROC partnerships shared many similarities across the four domains. However, worker cooperative partnerships often had unique aspects. Therefore, we report our findings for each domain separately for CLTs/ROCs and worker cooperatives. Also, given that the aim of the study is to inform healthcare organizations interested in collaborating with CWB organizations, the results mainly focus on the partnership aspects most relevant to the healthcare organizations, incorporating perspectives from both healthcare organization and CWB organization key informants.

### Motivation

#### CLT/ROC partnerships

Informants described motivations for the partnerships as deriving from both community needs and healthcare organization needs. Informants from every healthcare organization interviewed described housing unaffordability and/or gentrification-related displacement as primary motivators for the partnership. In several cases, Community Health Needs Assessments (CHNAs) played an important role in identifying housing security as an investment priority. As one healthcare organization informant described their CHNA result: “access to health care or chronic disease wasn’t at the top; it was housing.”

In this context, healthcare organizations saw CLTs/ROCs as well-suited for addressing various aspects of housing insecurity. Several healthcare organizations were drawn to the permanent affordability created by the CLT/ROC model in contrast to time-limited rental subsidies or traditional homebuyer programs that only benefit the first purchaser. Healthcare organizations also saw the homeownership aspect of CLTs/ROCs as important for “turning neighborhoods around” while preventing displacement. Finally, some saw CLTs/ROCs as an important racial equity intervention, as several of the CLTs/ROCs primarily served minoritized communities disproportionately excluded from traditional homeownership. One CLT/ROC organization informant described this priority of the healthcare organization: “The biggest thing they’re interested in right now is we’re reaching out to underserved, and more importantly, minority households underserved by homeownership projects in the past.”

In addition to community needs, healthcare organizations were also motivated by their own needs, what one informant described as “enlightened self-interest.” One key health system need was preserving affordable housing for their own workforce, which was also facing displacement. While none of the CLT/ROC partnerships focused exclusively on workforce housing, several targeted areas where lower-income staff members lived. One healthcare organization informant noted, “workforce housing need was a high motivator in socializing this [CLT/ROC] concept.” Another healthcare organization need was for good relationships with the local community. In some cases, CLT/ROC partnerships provided healthcare organizations the opportunity to demonstrate a commitment to respond to local problems like the housing affordability crisis. A healthcare organization informant observed an important motivator for the healthcare organization was, “seeing potential to improve the health of kids but also to build better relationships with the neighborhood.”

#### Worker cooperative partnerships

Worker cooperative partnerships were also motivated by community needs and healthcare organization interests. High rates of poverty and what one worker cooperative informant described as “a dire need for jobs and economic inclusion” were key community factors motivating worker cooperative partnerships. Worker cooperatives not only provided living wage jobs but also wealth-building opportunities as worker-owners share the firms’ profits. Two of the worker cooperative partnerships also explicitly sought to address racial wealth inequity by focusing cooperative efforts in minoritized communities.

Worker cooperative partnerships also addressed healthcare organization needs. In some cases, cooperatives provided local, reliable, and environmentally sustainable services not otherwise available within the hospital’s supply chain, such as energy-efficient laundry services. One healthcare organization informant described the worker cooperative partnership model as “local residents working at a local business providing a vital service we need for healthcare and building at the same time wealth for them.” Like CLT/ROC partnerships, worker cooperative partnerships also provided a means for the healthcare organization to strengthen its reputation in the community by channeling its purchasing power into local economic development and wealth-building.

### Initiation

#### CLT/ROC partnerships

While there were sometimes pre-existing collaborations between the healthcare organization and CLT/ROC organization on a non-CLT/ROC project, the formal initiation of the CWB partnership was generally catalyzed by a funding or investment opportunity initiated by the healthcare organization. Informants emphasized two major aspects of this initiation process for the healthcare organization: identifying partners outside the healthcare organization and obtaining “buy-in” within the healthcare organization.

In identifying community partners for their funding and investment opportunities, healthcare organizations sometimes specifically sought out CLTs/ROCs given their unique benefits. In other cases, healthcare organizations sought to partner with a particular community organization and only later learned of the CLT/ROC aspect of their work. In order to partner effectively with CLTs/ROCs, healthcare organizations often realized they also needed to involve additional community partners. These varied depending on the CLT/ROC’s internal capacities but sometimes included “underwriting partners” like community development financial institutions (CDFIs) as well as neighborhood associations or city housing departments to provide additional financing expertise, community involvement, or public resources and support.

In addition to identifying partners outside the healthcare organization, initiating the partnership also required obtaining buy-in within the healthcare organization. Most CLT/ROC partnerships were initiated by the healthcare organization’s community health leadership who then sought buy-in from the executive and/or finance team. Two main strategies were used to obtain buy-in: education and “risk mitigation.” Because CLTs/ROCs are relatively uncommon, educational meetings were set up between CLTs/ROCs and healthcare organization leadership to explain the benefits of the models and answer any questions. As one healthcare organization informant described, their process involved a “six-month period to understand what is a land trust,” in which the healthcare organization board, “had land trust folks come to a meeting to explain what a CLT is.” The uniqueness of the CLT/ROC models also raised concerns about risk, especially since almost none of the healthcare organizations knew of other healthcare organizations investing in CLTs/ROCs. One risk mitigation approach healthcare organizations took was making small, incremental investments and utilizing both grants and loans based on the community partner’s needs and capacity. Another risk mitigation approach was developing long-term relationships with CLT/ROC organizations, including having a healthcare organization representative on their board, to develop deeper trust and knowledge of their business operations.

#### Worker cooperative partnerships

As for the CLTs/ROCs partnerships, worker cooperative partnership informants also emphasized the importance of securing external partners and internal buy-in for initiating the partnership. The key external partner for worker cooperative partnerships was the worker cooperative developer organization, which could develop a new cooperative or connect the healthcare organization to an existing cooperative to meet a healthcare organization purchasing need.

As with CLT/ROC partnerships, worker cooperative partnerships also sought healthcare organization buy-in through education and risk mitigation strategies. In terms of education, informants from all three partnerships emphasized the crucial role visits with successful worker cooperatives played in getting buy-in, with one noting “a tour over two to three days is worth a year of talking.” Members of the more recent worker cooperative partnerships had the advantage of being able to visit the first worker cooperative partnership (University Hospitals and Cleveland Clinic with Evergreen Cooperatives), but given the novelty of their contexts and approaches, risk mitigation was still a priority. Informants described several risk mitigation strategies, including having a healthcare organization representative on the cooperative advisory board and converting existing businesses into worker cooperatives rather than starting a new business. In addition to these strategies, informants also emphasized the importance of specifically obtaining buy-in from the healthcare organization’s middle managers, who directly control purchasing decisions, in order to facilitate purchasing from the worker cooperatives. One worker cooperative informant explained, “just because the CEO says it doesn’t mean Joe in procurement will do it…part of the organizing strategy is organizing the C-suite leadership and also the procurement people.”

### Implementation

#### CLT/ROC partnerships

The implementation domain describes the “what” of the partnership, and informants specifically described what resources healthcare organizations provided in the partnerships and what roles they played. In terms of financial resources, healthcare organizations used grants and/or impact investments to support CLTs/ROCs. Table 2 provides a general description of the resources provided by healthcare organizations within each partnership.

**Table 2:** Characteristics of community wealth-building partnerships

Partnership	Investment Type	Healthcare Organization Role/Approach^a^
**Community Land Trusts (CLTs)**		
Bon Secours Richmond Health System – Maggie Walker Community Land Trust	Grant Impact Investment	Contributor Convener
Bon Secours St. Francis Health System – Sterling Land Trust	Grant Strategic Planning Support Technical assistance	Contributor Capacity-Builder
Mayo Clinic – First Homes Community Land Trust	Grant	Contributor
Nationwide Children’s Hospital – Central Ohio Community Land Trust	Grant	Contributor Convener
Saint Alphonsus Health System – LEAP Housing	Grant	Contributor Convener
UMass Memorial Medical Center – Worcester Common Ground	Impact Investment	Contributor
**Resident-Owned Communities (ROCs)**		
Dartmouth Health – ROC-NH	Impact Investment	Contributor
**Worker Cooperatives**		
Baystate Health – Wellspring Cooperative	Grant Purchasing Contract	Outside-in
Cleveland Clinic & University Hospitals – Evergreen Cooperatives	Grant Purchasing Contract	Outside-in
Kaiser Permanente – Obran & Project Equity	Grant Purchasing Contract	Inside-out

^a^Contributors provide support through financial capital (grants, impact investments, or purchasing contracts)

Conveners provide support through social capital (fundraising assistance, networking support, etc.)

Capacity-builders provide targeted assistance to develop the ROC/CLT entity

Outside-in refers to healthcare organizations bringing outside worker cooperatives into their supply chain

Inside-out refers to healthcare organizations helping convert existing suppliers into worker cooperatives

In terms of the overall roles healthcare organizations played in supporting CLTs/ROCs, a typology of three basic roles emerged among informants. Healthcare organizations acting as *contributors* provided financial capital to the CLT/ROC in the form of grants or loans. All the healthcare organizations studied acted as contributors. In most cases, healthcare organizations were the first major financial contributors to the CLT/ROC, which one CLT/ROC informant described as, “foundational for other investments.”

Some healthcare organizations also acted as *conveners*, using their social capital to bring together resources to support a CLT/ROC. A CLT/ROC informant noted their healthcare organization partner, “has a strong pull and when they come to a project they bring a lot of partners with them.” One healthcare organization led a broad fundraising effort for a CLT/ROC while another connected a CLT/ROC with partners to apply for tax credit funding.

Finally, one healthcare organization focused primarily on their role as a *capacity builder*, providing technical assistance, strategic planning resources, and other supports to help a CLT/ROC develop. One healthcare organization informant explained “I was able to get the hospital to fund strategic planning for the group and they need to figure out where they need to go.” This role can be especially important with a new or developing CLT/ROC.

#### Worker cooperative partnerships

Healthcare organizations supported worker cooperative partnerships primarily through purchasing from the worker cooperative (e.g. laundry services, solar installation, furniture upholstery) and also through grants aimed at cooperative capacity-building. Partnerships took one of two approaches to supporting worker cooperatives. Two healthcare organizations took *outside-in* approaches, which focused on bringing existing worker cooperatives into their supply chains. One healthcare organization took an *inside-out* approach, which focused on identifying businesses in its current supply chain to convert into worker-owned cooperatives. This healthcare organization helped fund two organizations with expertise in cooperative conversions to identify businesses interested in converting and support them through the conversion process.

### Evaluation

#### CLT/ROC partnerships

While informants referenced the established health benefits of housing stability, affordability, and wealth-building as important impacts of the partnerships, they also noted that capturing these impacts through return-on-investment (ROI) calculations or health outcome metrics would be challenging for two reasons. First, the impacts of wealth-building and neighborhood stability can take years or even decades to be fully realized, limiting the utility of shorter-term assessments. Second, many of the benefits of the CLT/ROC may go to people who are not healthcare organization patients or may improve patients’ health and well-being in ways that don’t primarily impact healthcare utilization. One informant contrasted the broad impacts of CLT investments with “surgical investments” aimed at reducing asthma emergency room admissions through interventions like mold abatement, which can show a positive return on investment. While acknowledging the value of both approaches, he stated, “we’re not approaching this from an ROI perspective. We’re doing neighborhood change.”

Rather than relying on health outcome measures, informants described the use of process measures to assess whether health-enhancing neighborhood changes were occurring. These include the scale of the project (e.g. number of CLT/ROC units), affordability (e.g. median home price, income of residents), neighborhood metrics (e.g. neighborhood vacancy rates), and wealth-building (e.g. net equity for sellers). Some partnerships are also tracking racial equity measures, including the racial demographics of the CLT/ROC and of the surrounding neighborhood. One health system reported a longer-term approach by measuring changes in the social vulnerability index over the next five to ten years in neighborhoods with CLTs and other healthcare organization investments.

#### Worker cooperative partnerships

Worker cooperative partnerships noted similar impact measurement limitations and also focused primarily on health-associated process measures. Those include scale (e.g. number of cooperatives and cooperative members), compensation (e.g. wages and benefits), wealth-building (e.g. dividends from cooperatives), and racial equity (e.g. racial demographics of cooperative members).

## Discussion

Among the diverse healthcare-CWB partnership cases studied, several common themes emerged within each domain. For healthcare organizations, a combination of community needs and their own needs motivated the partnerships, and initiating the partnerships required identifying external partners as well as securing internal buy-in through education and risk mitigation strategies. Healthcare organizations used their financial and social capital to play a variety of support roles across partnerships, and they sought ways to assess the benefits of the partnerships while acknowledging the limits imposed by long time-horizons and broad impacts.

**Fig. 1:**
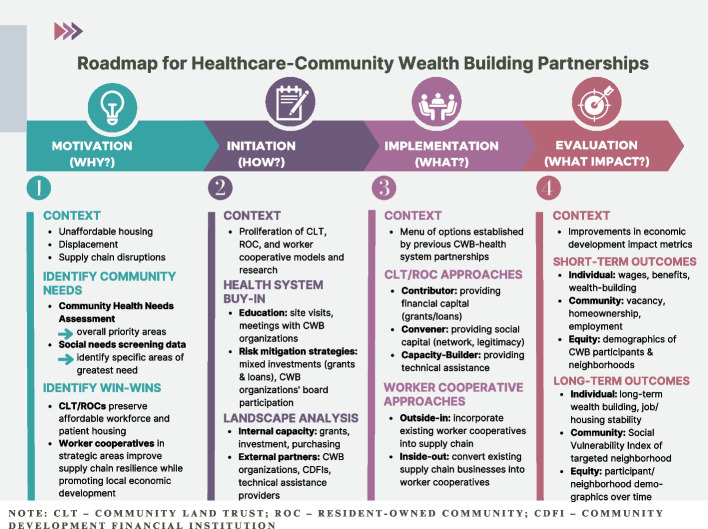
Roadmap for Health System Community Wealth Building Partnerships

As informants described in the Initiation domain, the vast majority of partnerships developed their models de novo, with little to no knowledge of the other partnerships. This is likely partly the result of timing, given many of these efforts were developed simultaneously, and partly due to the absence of systematic assessments of similar efforts. To inform future partnerships, this section begins by presenting a roadmap (Fig. 1), which synthesizes the findings from the multiple case study within the context of changes wrought by COVID and more recent developments in CWB interventions. After summarizing key implications for future partnerships within each domain of the roadmap, we review potential future directions for research that address limitations of the current analysis and describe broader public health implications of healthcare-CWB partnerships.

### Motivation

Addressing housing affordability and supply chain resilience were key motivators for developing the partnerships studied. Changes wrought by COVID and recent economic trends have only served to increase these motivations for healthcare organizations. Skyrocketing housing prices have made both homeownership and renting increasingly unaffordable across the country, and the expiration of eviction and foreclosure moratoria have left millions vulnerable to displacement [31]. COVID also exposed the vulnerability of health system supply chains, highlighting the need for more local and reliable suppliers [32].

These changes have created the possibility for even greater win-wins for healthcare organization partnerships with CLTs/ROCs and worker cooperatives. The permanent affordability and stability of CLTs/ROCs provide a unique remedy to the rampant displacement impacting not only healthcare organizations’ patients but also their staff. In addition, healthcare organization partnerships with worker cooperatives can increase local supply chain resilience while also supporting community economic development and individual wealth building.

Finally, designing win–win partnerships requires a regular assessment of the local community’s evolving needs. In addition to the Community Health Needs Assessments several informants used to inform CWB partnerships, healthcare organizations can also use patient social needs data they are increasingly collecting to more specifically target neighborhoods and populations with the greatest need [3].

### Initiation

Key aspects of the initiation phase identified by informants – including identifying external partners and securing healthcare organization buy-in – can benefit from the proliferation of CWB models and growing evidence of their impacts in recent years. The number of worker cooperatives in the US grew 30% between 2019 and 2021, [23] while ROC USA – the largest national network of resident-owned communities – has more than doubled in size over the past decade [16]. Several recent analyses have further elaborated the economic benefits associated with these models [12, 33–36]. For healthcare organizations, this means the initiation phase is now supported by a broader network of potential partners and a deeper pool of accumulated experience.

In addition, the major healthcare organization resources informants identified in the implementation phase (e.g., grants, investments, purchasing) can also inform the initiation phase. In addition to identifying good external partners, healthcare organizations can also assess what internal resources they can bring to the partnership through a broader “landscape analysis” of both their internal and external environments. This could help healthcare organizations to see beyond the specific funding, investment, or purchasing opportunity that initiated the partnership to a broader set of potential resources and collaborators that could strengthen the partnership.

### Implementation

Because the partnerships interviewed were diverse in their locations and approaches, there are now several established paths healthcare organizations can choose. Based on their landscape analysis, healthcare organizations can consider to what extent to play a contributor, convener, and/or capacity-builder role with CLTs/ROCs. For CLTs ready to purchase properties, being a contributor as a first investor could play a catalytic role in bringing other funders to the table. The health system could also play a convening role by promoting CLTs/ROCs as a strategy among local housing providers and funders. If there are no established CLTs/ROCs locally, the healthcare organization can play a capacity-building role by contributing grant funds for a feasibility study or strategic planning for a potential CLT/ROC host organization. Similarly, healthcare organizations can also use their landscape analysis to determine whether to pursue a worker cooperative partnership strategy that is outside-in or inside-out, depending on whether there is a local cooperative providing relevant services or a current supplier interested in converting into a cooperative.

### Evaluation

The types of evaluation metrics described by informants can be divided into short-term and long-term metrics focusing on individuals, communities, or equity. Figure 1 elaborates different examples of metrics that could be included in each category. Metrics to assess the impacts of economic development interventions are increasingly being developed and can be useful for evaluating CLT, ROC, and worker cooperative partnerships [37]. While these metrics cannot completely isolate program effects from other factors influencing outcomes, they provide a more comprehensive picture of the partnerships’ impacts across time and population that can guide future program design.

### Limitations and directions for future research

This analysis has several limitations. Our sample did not include CWB partnerships that were attempted but did not lead to healthcare organization investments, and there are likely important lessons to learn from these unsuccessful attempts not captured in our analysis. Also, given that the informants were often reporting about past events, their responses may be subject to recall bias. In addition, many of the programs had not yet begun the evaluation phase, especially around long-term outcomes, so their responses in this area may be more reflective of plans and aspirations than actual practice. Finally, while the healthcare organizations interviewed represent significant diversity in terms of geography, community demographics, and partner landscape, it is unclear the extent to which these findings can be generalized to settings such as for-profit healthcare organizations, small community hospitals, or settings outside the US. Additional studies including unsuccessful partnerships, data collection concurrent with each stage of the partnership, non-US partnerships, or other hospital types could help provide a clearer picture of the challenges, impacts, and applicability of these types of partnerships.

### Public health implications

Community wealth building strategies like CLTs, ROCs, and worker cooperatives provide health care organizations an opportunity to transform their housing investments and purchasing practices in ways that have impact further upstream – promoting housing and job security in their communities. One-off housing or income supports, while important, cannot do this.

Importantly, evidence suggests the benefits of these models aren’t limited to the CLT/ROC residents or the worker cooperative members alone. By building wealth and stability, these models promote economic resilience among entire communities and prevent displacement. For example, foreclosures produce well-documented harms to neighborhoods and community health, [38] and CLTs have been shown to reduce foreclosures by as much as 90% [13]. Beyond protection from foreclosures, CLTs have also been shown to reduce gentrification-related displacement at the neighborhood level [35]. Similarly, worker cooperatives provide stable employment and local spending that can promote broader community economic development [39]. Regions with a high density of worker cooperatives have been especially resilient to recessions, laying off less than one percent of their workforce while comparable regions experienced mass layoffs during the Great Recession [25]. Given their participant- and community-level impacts, scaling up these CWB models could have immense public health benefits.

## Conclusion

Community wealth building represents a new paradigm of upstream healthcare approaches to the social determinants of health. This systematic assessment of healthcare organization partnerships with community wealth building organizations reveals common themes and key lessons. Applying these lessons to the present context provides a roadmap that other healthcare organizations can follow to develop more effective partnerships in their efforts to improve the health of their patients and their communities.

## Data Availability

The datasets used and/or analysed during the current study are available from the corresponding author on reasonable request.

## Abbreviations

CDFI: Community development financial institution
CHNA: Community health needs assessment
CLT: Community land trust
CWB: Community wealth building
HAN: Healthcare Anchor Network
ROC: Resident-owned community
ROI: Return on investment
