# Correction to: Moving upstream: healthcare partnerships addressing social determinants of health through community wealth building

**DOI:** 10.1186/s12889-023-17054-z

**Published:** 2023-10-30

**Authors:** Geoffrey M. Gusoff, David Zuckerman, Bich Ha Pham, Gery W. Ryan

**Affiliations:** 1 National Clinician Scholars Program & Department of Family Medicine, University of California, Los Angeles, 1100 Glendon Ave, Suite 900, Los Angeles, CA 90024 USA; 2 Healthcare Anchor Network, 2202 18th St. NW, Suite 317, Washington, DC 20009 USA; 3 Department of Health Systems Science, Kaiser Permanente Bernard J. Tyson School of Medicine, 100 South Los Robles Avenue, Pasadena, CA 91101 USA

**Correction to: BMC Public Health 23, 1824 (2023).**

10.1186/s12889-023-16761-x

The original publication of this article contained an affiliation error, the correct and incorrect information is listed in this correction article. The original article has been updated.

**Incorrect**

- Bich Ha Pham^3^
- ^3^Department of Health Systems Science, Kaiser Permanente Bernard J. Tyson School of Medicine, 100 South Los Robles Avenue, Pasadena, CA 91101, USA.

**Correct**

- Bich Ha Pham^2^
- ^2^Healthcare Anchor Network, 2202 18th St. NW, Suite 317, Washington, DC 20009, USA.

